# Building new capacity for Africa CDC through *The Journal of Public Health in Africa*

**DOI:** 10.4102/jphia.v15i1.679

**Published:** 2024-07-22

**Authors:** Nicaise Ndembi

**Affiliations:** 1 Africa Centres for Disease Control and Prevention, Addis Ababa, Ethiopia

As the first quarter of the 21st century draws to a close, Africa finds itself balancing the bright promise of its developmental aspirations with the realities of many challenges. Not least among these is the need to strengthen its health systems on many fronts, including increasing capacity to fight concurrent, interacting epidemics – often referred to as ‘syndemics’. The term syndemics encapsulates the theory that multiple epidemics occurring at the same time and in the same region will interact at both the individual and population levels, resulting in worse outcomes than any one epidemic alone.^1^ As a continental community, Africa certainly has its fair share of opportunities to test this theory.

The continent is faced with a set of linked health problems that interact synergistically, contributing to its excess burden of disease: rising rates of non-communicable diseases (NCDs), emerging and reemerging infections and endemic diseases. The proportion of NCD-related deaths increased from 24% in 2000 to 37% in 2019, highlighting the increasing challenges of NCDs in Africa.^2^ By 2030, NCDs are expected to outpace all other disease types as the leading cause of mortality. Despite many gains over recent decades, infectious diseases still have a severe impact on the African continent, accounting for over 227 million years of healthy life lost every year and producing an annual productivity loss of over $800 billion. Multiple factors are contributing to the rise of emerging infectious diseases in Africa, with more than 100 outbreaks reported annually.^3^ The world has also entered a new era of infectious disease threat because of climate change, the destruction of animal habitats, human encroachment into previously isolated areas and increasing interactions between people and animals, thereby fuelling spillover events and accelerating the emergence of new-to-humans diseases.^4^

The Africa Centres for Disease Control and Prevention (Africa CDC) has worked diligently as it was launched in January 2017 to position itself to meet these challenges. In 2023, Africa CDC’s new Advisory and Technical Council and the constitution of the new Governing Board were established and inaugural meetings established key strategic milestones. These included the approval of the 2023–2027 strategic plan for Africa CDC and a strategic reorientation of the Saving Lives and Livelihoods initiative. This partnership between Africa CDC and Mastercard Foundation is supported by a $1.5 billion grant and focuses on the vaccination of high-risk populations, integration of COVID-19 response into routine immunisation and primary healthcare and proactive preparation for potential future pandemics on the continent.^5^ An additional milestone is the approval of the African Vaccine Manufacturing Accelerator (AVMA) and implementation of Partnerships for African Vaccine Manufacturing (PAVM) strategic priorities with a focus on vaccine manufacturing and supply chain capabilities in the continent. Africa CDC has also developed strategic priorities for NCDs in Africa, including enhancing capacity to develop and implement policies to prevent, protect and manage NCDs; political advocacy for better financing and related workforce development and increased access to essential technologies, medicines and diagnostics in Africa.

As Africa CDC moves to implement the 2023–2027 strategic plan, efforts are underway to ensure that Africa CDC is adequately equipped and staffed with the required technical expertise to deliver on its mandate at the continental, regional and country levels. Internal mechanisms are in place to empower our staff, offer training and career growth opportunities and provide them with the essential support they need. As part of these efforts, Africa CDC is investing in its scholarly, peer-reviewed journal, the *Journal of Public Health in Africa* (JPHIA) to ensure that African research has a home with a 50% article processing charges (APC) subsidy and a scientific manuscript writing programme to support young investigators across the continent.

The *Journal of Public Health in Africa* became the official journal of Africa CDC in 2018 after reaching an agreement with PAGEPress, its original publisher, whereby Africa CDC oversaw the journal’s editorial and peer-review processes. The *Journal of Public Health in Africa* received a Journal Impact Factor from Web of Science in 2023, and in 2024, began a year of transformation, first by transitioning JPHIA to an Africa-based, open-access publisher, AOSIS. A rejuvenation of the JPHIA Editorial Board is also being undertaken in 2024, and a number of esteemed, international scientists and experts from across the African continent and beyond have joined the Board to provide editorial advice and support to the journal until 2027. Furthermore, a geographically diverse and multidisciplinary Editorial Team was formed, also serving until 2027, to handle the behind-the-scene, day-to-day work of the journal. The Editorial Team comprises of JPHIA’s Section Editors who specialise in 15 broad areas (Box 1). These multitalented teams of African and global scholars, practitioners and researchers – along with the authors and reviewers that will contribute to the journal – will be important in supporting Africa CDC’s goals and mission through the publication of the high-quality research and commentary that is published in JPHIA in the coming years.

BOX 1 *Journal of Public Health in Africa* sections.

**Sections:**

- Ageing and Health Promotion
- Biostatistics and Bioinformatics
- Cardiovascular Diseases
- Data Science
- Epidemiology
- Governance and Leadership
- Health Care and Policy (Global Health)
- Infectious Diseases
- Maternal, Newborn, and Child Health (MNCH)
- Mental Health and Injuries
- Microbiology, Immunology and Vaccinology
- One Health Approach (Human, Animal, and Environment)
- Pandemic Preparedness
- Recent Trends in Traditional Medicine, Health Research and Development
- Virology

As outlined above, there is much to do on many fronts to achieve Africa’s aspirations for its health sector. For instance, there is still much hard work ahead to recover from the pandemic-related health service delivery and access disruptions. This presents an opportunity to incorporate some of the innovations adopted to sustain services at the height of the COVID-19 pandemic and stretch the reach of services into the future. In addition, historical modelling suggests that the frequency and severity of epidemics caused by wildlife zoonoses – driven by human activities and their environmental impact – are increasing. Such modelling estimates that the probability of a future zoonotic-spillover event resulting in a pandemic of COVID-19 magnitude or larger is between 2.5% and 3.3% annually.^6^ In other words, there is a 22% – 28% chance that another outbreak of the magnitude of COVID-19 will occur within the next 10 years and a 47% – 57% chance that it will occur within the next 25 years.^7^

The public health issues detailed above must be researched, reported and shared in a quality scholarly publication in order to be addressed. This will, in turn, foreground such public health issues while simultaneously reducing and addressing those issues for the benefit of the African content and the *people* who live on it. Our aim is to position JPHIA as the journal of choice for the publication of Africa-related health research on these issues and more. We hope you will join us.
